# An Sp1 Modulated Regulatory Region Unique to Higher Primates Regulates Human Androgen Receptor Promoter Activity in Prostate Cancer Cells

**DOI:** 10.1371/journal.pone.0139990

**Published:** 2015-10-08

**Authors:** Colin W. Hay, Irene Hunter, Alasdair MacKenzie, Iain J. McEwan

**Affiliations:** Institute of Medical Sciences, University of Aberdeen, Foresterhill, Aberdeen, United Kingdom; Innsbruck Medical University, AUSTRIA

## Abstract

Androgen receptor (AR) mediated signalling is necessary for normal development of the prostate gland and also drives prostate cancer (PCa) cell growth and survival, with many studies showing a correlation between increased receptor levels and therapy resistance with progression to fatal castrate recurrent PCa (CRPC). Although it has been held for some time that the transcription factor Sp1 is the main stimulator of AR gene transcription, comprehensive knowledge of the regulation of the AR gene remains incomplete. Here we describe and characterise in detail two novel active regulatory elements in the 5’UTR of the human AR gene. Both of these elements contain overlapping binding sites for the positive transcription factor Sp1 and the repressor protein pur-α. Aberrant cell signalling is characteristic of PCa and the transcriptional activity of the AR promoter in PCa cells is dependent upon the relative amounts of the two transcription factors. Together with our corroboration of the dominant role of Sp1, the findings support the rationale of targeting this transcription factor to inhibit tumour progression. This should be of particular therapeutic relevance in CRPC where the levels of the repressor pur-α are reduced.

## Introduction

Prostate cancer (PCa) is the second most prevalent cancer in men and constitutes the second leading cause of male cancer deaths in Western nations and the sixth worldwide respectively [1]. In addition, the incidence of PCa is rising in virtually all countries with the rate of new cases expected to double by 2030 to 1.7 million resulting in 500,000 additional deaths [2].

The growth and differentiation of normal prostate epithelial cells, as well as development and progression of PCa, are driven by androgen signalling which is mediated by the androgen receptor (AR) [3]. Therefore, inhibition of AR function by androgen deprivation therapy (ADT) using antagonists or abatement of testicular or intratumoural androgen synthesis constitutes the principal procedure for tackling advanced and metastatic PCa [4–6]. Initially, most patients experience significant improvement and remission, but the cancer invariably evolves to become independent of circulating androgens and is referred to as castrate resistant PCa (CRPC) [7]. This late, metastatic stage is usually fatal and represents the greatest challenge for the development of new, efficacious therapies [8]; necessitating multiple AR-targeted regimens (reviewed in [9]). Crucially, CRPC tumours remain dependent upon AR signalling; exemplified in both androgen sensitive (AS) and CRPC cells where decreased AR expression induces a concomitant loss of cell viability [10].

The androgen receptor functions as an androgen-activated transcription factor that binds to androgen response elements (AREs) [4] in promoters and distal enhancers (86% to 95% of AREs [11]). The transactivation capability of the receptor is modulated by interactions with an ever-growing list of coregulators and transcription factors (reviewed in [12]) that act on the receptor itself or alter the chromatin environment. For example, the pioneer transcription factors FOXA1 and GATA2 promote an open chromatin structure that facilitates AR binding, and genome wide studies show co-localisation of binding sites for these three factors [13,14]. GATA2 plays a particularly important role because as well as increasing AR binding to enhancers, it participates in chromatin looping and directly upregulates AR gene expression [13,14]. Elevated levels of GATA2 in PCa correlate with high Gleeson scores, and reduced activity through dampened expression [13,14] or inhibition of GATA2 occupancy at AREs with the isoflavone curcumin [15] result in lower PCa cell proliferation.

The majority of CRPC tumours overexpress the receptor [16–19] with clonal selection exacerbating the problem [20]. The elevated levels of AR permit binding to chromatin in 100-fold lower concentration of ligand than normal [21] and the aberrant AR-driven transcriptional programme in CRPC tumours allows cells to grow in low concentrations of androgen [22] or the apparent absence of hormone [23–25], thereby abrogating ADT [26] and treatment with abiraterone [20].

In humans, the AR gene spans approximately 180 kbp of the X-chromosome (Xq11.2-q12) and gives rise to a transcript of 4.3 kb that includes an uncommonly long 5’ untranslated region (5’UTR) of 1.1 kb. The promoter lacks TATA and CCAAT boxes and, in common with many TATA-less genes, transcription is driven primarily by binding of the ubiquitously expressed zinc finger transcription factor, Specificity Protein 1 (Sp1) to GC box regulatory elements. The core promoter lies between -74 and +87 bp [27] and active GC boxes have been confirmed at -46 to -41 bp as well as in the 5’UTR at +429 and +442 bp [27–29]. Sp1-driven expression of the AR gene is facilitated by an approximately 90 bp stretch of homopurine/homopyrimidine immediately upstream of the promoter (-150 to -60 bp) that provides an abundant supply of the transcription factor to bind to the core promoter [30]. Once bound to the promoter, Sp1 associates directly with the TATA-binding protein and TBP-associated factor 4 (TAF4) [31] to establish the initiation complex. In addition, Sp1 can form multimers and multiple stacked tetramers providing numerous docking sites for a variety of other proteins to regulate transcription both directly and through histone acetylation and chromatin remodelling (reviewed in [32]). In addition to the upregulatory GC boxes, the 5’UTR region encodes several inhibitory regulatory elements including a composite NF-κB, B-myb binding site [33], an negative ARE [34] and an Androgen Receptor Suppressor (ARS) [35] that binds pur-α and hnRNP-K on opposite strands of the DNA [36].

The AR clearly represents the Achilles’ heel of prostate cancer yet the current primary treatment involving androgen antagonists has limitations and also leads to expression of an alternative AR-driven transcriptome with variable PCa outcomes [37]. A much more efficacious approach would be to reduce AR activity through reduced expression or interference with essential transcription cofactors e.g. a small molecule inhibitor of GATA2 has proven to be effective [13]. Both approaches depend upon detailed knowledge of the interactions of the multiple transcription factors and regulatory elements involved in AR expression. Therefore, as the transcription factor Sp1 is generally considered to be a major stimulator of AR gene expression, we have studied Sp1 function at the immediate promoter and 5’UTR of the human AR gene. In this report we describe two novel active regulatory elements in the human AR 5’UTR that both contain overlapping binding sites for positive and negative transcription factors. The transcriptional outcome in PCa cells is dependent upon the relative amounts of stimulatory Sp1 and inhibitory pur-α. The findings also substantiate the dominant role of Sp1 in driving AR expression and thus reinforce the rationale of targeting this transcription factor, as this action will promote binding of competing pur-α and inhibit tumour progression. Lastly, the regulatory elements exhibit very poor evolutionary conservation illustrating the distinctive nature of human AR gene regulation.

## Materials and Methods

### Cell culture

Human prostate carcinoma cell lines LNCaP and DU145 were obtained from The European Collection of Cell Cultures and the American Type Culture Collection respectively. DU145 were grown in DMEM while LNCaP were maintained in RPMI containing 1 mM Na pyruvate and 10 mM HEPES. All media were supplemented with 10% foetal bovine serum (PAA) and maintained at 37°C without antibiotics in a humidified atmosphere containing 95% air and 5% CO_2_.

### Western blotting

Cell extracts were prepared from LNCaP and DU145 cells, and western blots performed as detailed previously [38]. Human Sp1, pur-α, hnRNP-K, and GAPDH were detected using antibodies ab13370, ab79936, ab52600 and ab36840 (all from Abcam) at dilutions of 1:6,000, 1:60,000, 1:17,000 and 1:12,500 respectively. Anti-AR from Santa Cruz Biotechnology (sc–7305) was used at 1: 100 dilution. Antigen-antibody complexes were detected using horseradish peroxidase-conjugated anti-rabbit IgG secondary antibody (Sigma) as previously described. Integration analysis of western blots was carried out using Image J software.

### RT-PCR

LNCaP cells were treated with DMSO or 50 nM mithramycin A (Sigma) for 24 hours. Extraction of RNA and RT-PCR for AR and GAPDH mRNA were carried out as described previously [34].

### Plasmids and site directed mutagenesis

Mutations of potential regulatory elements were introduced into plasmid phAR1.6Luc, in which luciferase expression is driven by 1.6 kbp of the promoter and 5’UTR of the human androgen receptor gene (between -741 and +842 bp) [34], using two methodologies. Base substitutions were created using the QuikChange II Mutagenesis kit (Agilent Technologies) using the oligonucleotides listed in Table A in S1 File, along with their reverse complements. Deletion mutations were made using the In-Fusion Advantage PCR cloning system from Clontech using the oligonucleotides listed in Table B in S1 File. Both protocols were performed according to the manufacturer’s protocol and the integrity of all constructs was confirmed by DNA sequencing.

### Transfection and Luciferase reporter gene assays

Twenty four-well plates were seeded with LNCaP or DU145 cells at a density of 5 x 10^4^ cells/cm^2^ and 1.2 x 10^4^ cells/cm^2^ respectively. The cells were cultured in complete medium for 24 h, then transfected with 440 ng/well of firefly luciferase reporter plasmid using JetPEI polyethylenimine (Polyplus Transfection) according to the manufacturer’s protocol. After 24 h, the medium was replaced and the cells were cultured for a further 24 or 48 h.

Plasmid transfection was performed in at least triplicate and luciferase activity was measured in duplicate or triplicate by using a GloMax 96 Microplate luminometer (Promega) and normalised for protein concentration as previously described [38].

### Preparation of nuclear extracts

Nuclear extracts were prepared from DU145 cells in the presence of protease inhibitors (complete protease inhibitor cocktail from Roche plus 1.0 mM PMSF) and protein phosphatase inhibitors (5 mM β-glycerophosphate and 100 μM activated Na_3_VO_4_) using the method of Dignam *et al* [39].

### Electrophoretic mobility shift assays (EMSAs)

Either 10 μg LNCaP cell nuclear extract or 120 nM purified recombinant human Sp1 protein (Active Motif) were incubated with 20 fmol biotin 3’ end-labelled double stranded DNA oligonucleotides using previously described conditions [34]. The forward sequences of the oligonucleotides were: Sp1-1, 5’-CGGCCCGGTGGGGGCGGGACCCGACTCGCA–3’; ARS, 5’-TCTCCACCCCGCCTCCCCCCACCCTGCCTT–3’; and Sp1-3, 5’-TTCTCCCCACCCGCCCCCCCGCCCCCGTCG–3’. An unlabelled oligonucleotide of a consensus Sp1 regulatory element, Sp1 cons, 5'-ATTCGATCGGGGCGGGGCGAGC–3' was added 15 min prior to the labelled probe. For supershift assays the antibodies ab13370 and ab52600 (Abcam) against human Sp1 and hnRNP-K respectively were added 15 min prior to the addition of labelled probe.

The resulting DNA:protein products were resolved in cooled 6% nondenaturing polyacrylamide gels run in 0.5x TBE buffer, pH 8.3 (45 mM Tris-borate, 1 mM EDTA) and detected using Pierce LightShift Chemiluminescent reagents (Thermo Scientific) according to the manufacturer’s protocol. Figures were compiled using autorads of EMSA gels with the order of lanes within some gels being altered to aid clarity and facilitate comparisons. Digital integration of the DNA:protein complexes was carried out using a Vilber Loumat Fusion SL cooled CCD sensor with care being taken to ensure that no pixel saturation occurred.

### Chromatin immunoprecipitation (ChIP) assay

A detailed account of the ChIP methodology has been presented previously [34]. In brief, LNCaP cells were transfected with the relevant phAR1.6Luc-based plasmid and grown in the usual manner. Cells were fixed in 1% formaldehyde for 10 min at 37°C and nuclei were prepared. Chromatin and plasmid were digested with 200 units HphI (NEB) for 20 min at 37°C; followed by lysis and the removal of insoluble debris by centrifugation. The supernatant was diluted in ChIP buffer and precleared using Protein-G and Protein-A Dynabeads (Life Technologies). Samples of cleared lysates were retained as input (IP) and the remainder was incubated with antibodies against either Sp1 (07–645, Millipore) or IgG. Immunocomplexes were collected by magnetisation, washed twice each with: low salt; high salt; LiCl and TE buffers, followed by elution. DNA-protein cross-links were reversed with NaCl at 65°C and DNA purified. Isolated DNA was quantified by semi-quantitative log phase PCR and resolved by agarose gel electrophoresis in TAE buffer. The forward (F) and reverse (R) primers were: ARS-F, 5’-GCTGCTAAAGACTCGGAGGA-3’; ARS-R, 5’-CTGAGAGTAGCCGACTGAGG-3’; Sp1-3-F, 5’-CCCGAGTTTGCAGAGAGGTA-3’ and Vect-R, 5’-TCTTCCATGGTGGCTTTACC-3’.

### Statistical analysis

The statistical significance of differences in data sets of DNA:protein complex formation in EMSA experiments was determined using two way ANOVA and paired t-test analysis of variance was employed for all other comparisons between complementary data.

## Results

### The human AR gene 5’UTR contains two regions with overlapping regulatory elements

**Fig 1:**
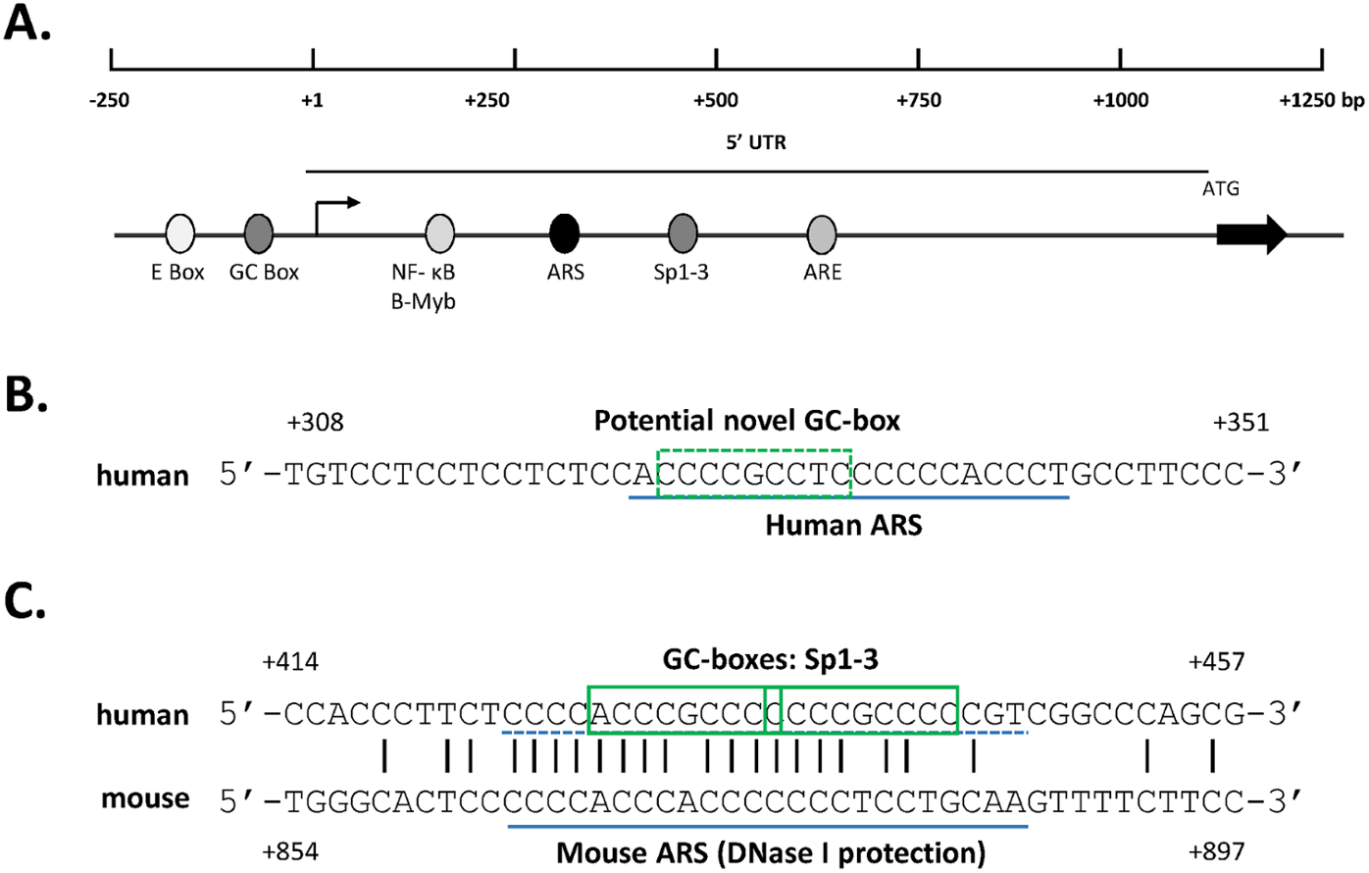
Regulatory elements in human AR 5’UTR. (A) Diagrammatic representation of the human AR gene 5’UTR and immediate proximal promoter showing the principal regulatory elements and the regions under study. Bent arrow indicates the transcriptional start site (+1) and ATG with solid arrow show the start of translation. (B) Potential GC box (green dashed box) within the confirmed human ARS regulatory element (blue underlined). (C) Alignment of the human AR 5’UTR and the mouse AR 5’UTR suppressor element protected from DNase I digestion [40] (blue underlined) with the confirmed human GC boxes (green solid box). Homologous sequences are indicated by vertical lines.

Examination of the human AR gene encoding the 5’UTR region of the transcript (Fig 1A) revealed a potential Sp1-binding GC box (+328 to +332 bp) that was found to lie within the previously described Androgen Receptor Suppressor (ARS) sequence [35] (Fig 1B). The first example of an ARS regulatory element that can bind the ubiquitously expressed protein pur-α was described in the mouse AR gene 5’UTR over 400 bp downstream from the corresponding human sequence [40]. As both GC boxes and ARS sites have high GC content, the possibility of other examples of these two regulatory elements overlapping was investigated further. Although binding of pur-α is sequence specific with a preference for repeats of (GGN), there is no clear consensus recognition sequence, therefore, the region of the mouse ARS protected from DNase I digestion was used to search for other possible sites in the human 5’UTR. A region of the human 5’UTR, distal to the ARS sequence, possessing 85% identity was detected (Fig 1C) which is greater than the 70% identity seen with the same probe and the established human ARS (S1A Fig). Use of the defined mouse ARS sequence [41] produced a similar finding with 75% identity which, again, was greater than the value of 67% for the human ARS (S1B and S1C Fig) respectively. The potential novel suppressor region overlapped the Sp1-3 regulatory element that is known to harbour two active GC boxes [29]. This raised the intriguing possibility that these two regions can act to either stimulate or reduce AR expression.

**Fig 2:**
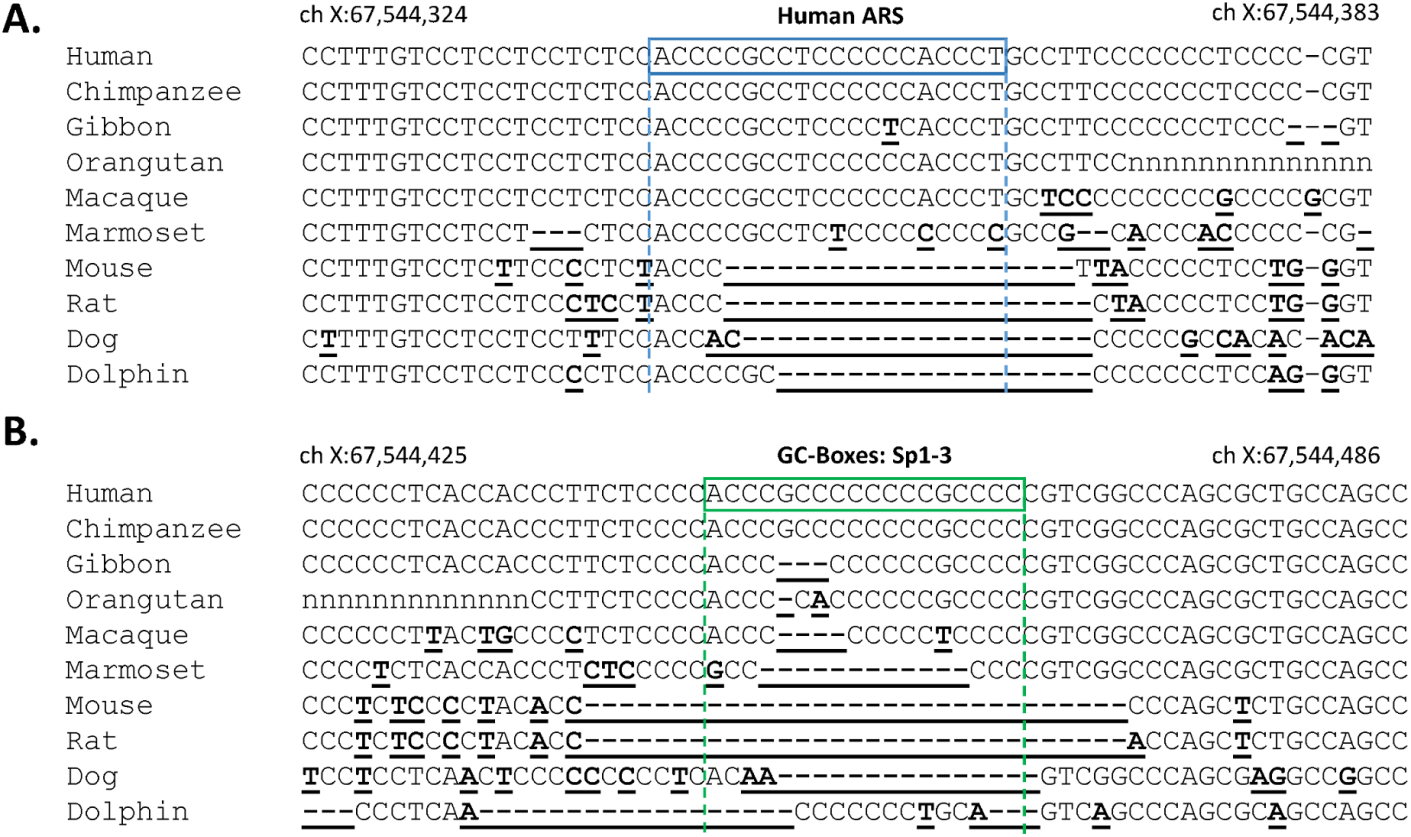
Multiple genome alignments of the AR 5’UTR. The regions of the hAR gene 5’UTR under investigation were compared to those of the indicated placental species. (A) the ARS (blue box), and (B) the Sp1-3 regulatory element (green box). Differences from the human sequence are indicated by bold, underlined font.

Multiple alignments of the equivalent regions of the human AR gene 5’UTR in 11 other species using publically available DNA sequences (Fig 2) showed that both sequences are poorly conserved. The ARS region is present only in primates and the sequence in chimpanzee, which diverged from humans 6.6 million years ago [42], has perfect homology with human. Furthermore, and over the span of 42.2 million years from the divergence of humans and marmoset, the most diverged primate examined, the majority of sequences show only a few nucleotide substitutions. The Sp1-3 region displayed even less homology with only chimpanzee sharing both GC boxes with humans. For both regions of interest, non-primate species possessed very low levels of homology with human, and no equivalent sequences were found in avian or piscine species (data not shown). In addition, the mouse ARS region is extremely poorly conserved and no pur-α binding sequence has been found in the equivalent genomic region of other animals including the closely related rodent; the rat (S2 Fig).

### The GC box is the principal Sp1 binding site in the human AR promoter

**Fig 3:**
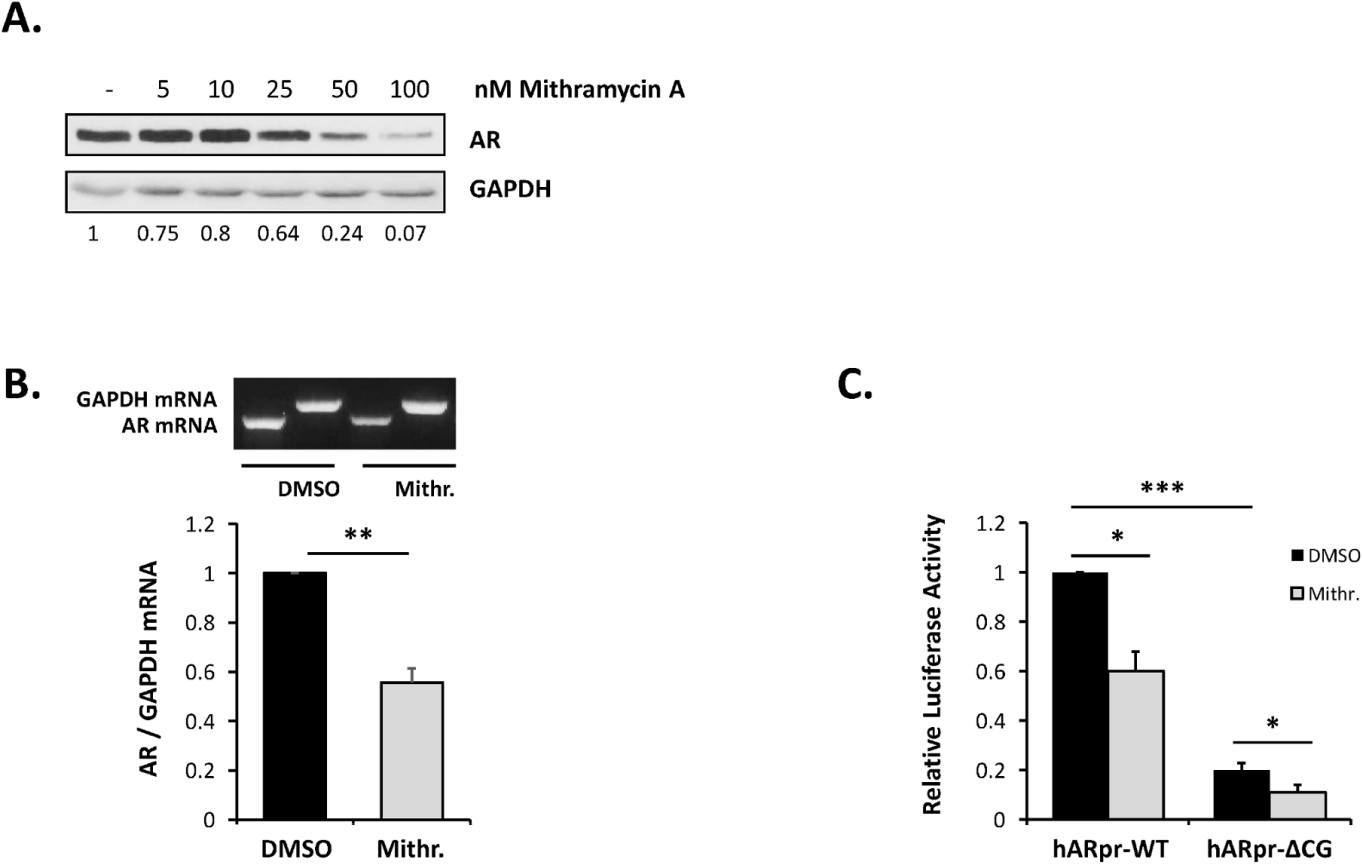
Effect of mithramycin A on AR mRNA and protein expression. LNCaP cells were incubated with mithramycin A for 24 h and then assayed. (A) Western blot analysis with the relative values of AR/GAPDH shown below. (B) RT-PCR of AR and GAPDH mRNA. LNCaP cells were transfected with phAR1.6Luc (WT) or phAR1.6Luc-ΔCG and treated with DMSO or 50 nM mithramycin A for 24 hours and luciferase activity was measured. Data represent the means ± SD of at least three independent experiments and the statistical significance of the indicated comparisons are: * p<0.05; ** p<0.01 and *** p<0.001.

Initial experiments examined the effect of the Sp1 antagonist, mithramycin A, on the endogenous AR gene in LNCaP cells. A mithramycin A dose dependent reduction in AR protein levels was observed (Fig 3A) with a concentration of 50 nM mithramycin A being used in subsequent experiments. RT-PCR studies confirmed that the decrease in AR protein levels correlated with reduced transcription (Fig 3B).

While it has long been held that the transcription factor Sp1 is the main driver of expression of the TATA box-lacking human AR gene, the relative importance of the GC box (Sp1-1; -45 to -40 bp) in the core promoter remains unclear with deletion studies suggesting that it is either essential [27] or does not play a significant role [43]. Mutation of the GC box in the phAR1.6Luc luciferase reporter plasmid [34] that contains a 1.6 kbp section of the hAR promoter and 5’UTR between positions -741 bp to +842 bp led to a 80% reduction in luciferase expression in transfected androgen responsive LNCaP cells (Fig 3C). Similarly, a 46% reduction was observed with the androgen non-responsive PCa cell line DU145 (data not shown). Together, these assays demonstrated that the core promoter GC box, and Sp1 binding, play a prominent role in the expression of AR mRNA and protein. Importantly, the mutated reporter plasmid remained susceptible to inhibition by mithramycin A (Fig 3C), thus confirming that Sp1-mediated upregulation of the AR gene occurs at sites other than the GC box.

### The 5’UTR overlapping regulatory elements can up- or downregulate promoter activity

In order to elucidate the functional activity of other potential regulatory elements under study, further mutations were introduced into the phAR1.6Luc luciferase reporter plasmid. Transfection experiments were performed using both androgen responsive, LNCaP and non-responsive, DU145 PCa cell lines, that were cultured in complete medium to ensure that all cell signalling pathways that are dependent on media components (growth factors and hormones), were functional.

**Fig 4:**
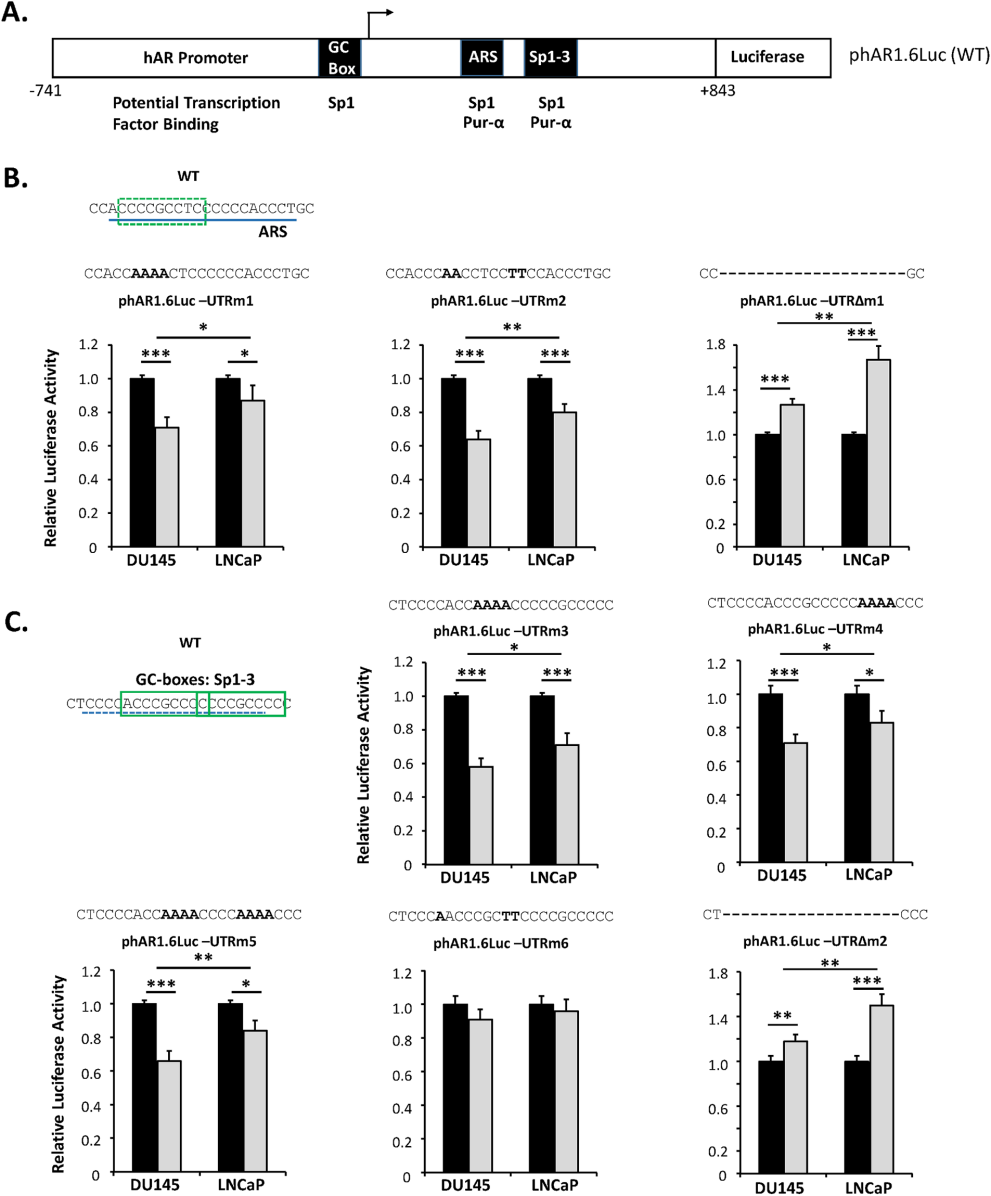
Effect of mutations on transcriptional activity. (A) Schematic diagram of the luciferase reporter construct phAR1.6Luc driven by 1.6 kbp of the hAR promoter and 5’UTR with potential transcription factor binding to the regions of interest. Sp1-3 contains two Sp1 binding sites. (B and C) The indicated PCa cell lines, cultured in complete medium, were transfected with either phAR1.6Luc containing the WT sequence (black bars) or the mutated version (grey bars) shown above each chart. Luciferase data represent the means ± SEM of at least three independent experiments and the statistical significance of the indicated comparisons are: * p<0.05; ** p<0.01 and *** p<0.001.

In order to establish the role of Sp1 binding to the potential GC box in the ARS (+322 to +345 in the 5’UTR), base substitutions were introduced to create reporter phAR1.6Luc-UTRm1 that resulted in reductions of expression of 29% and 13% in DU145 and LNCaP cells respectively (Fig 4A and 4B) confirming an active role for this novel GC box in upregulating AR expression. Conversely, this region of the 5’UTR has been shown to act as a negative regulatory element through the binding of the transcription factor pur-α that has been reported to be disrupted under EMSA conditions by the inclusion of a pair of two-base substitutions [35] and these were used to prepare reporter phAR1.6Luc-UTRm2. However, using this reporter transcriptional activity was reduced by 36% and 20% in DU145 and LNCaP respectively rather than increased (Fig 4B) as might be predicted upon release of an inhibitor. Inspection of the sequence changes produced by the mutation reveals that the guanine and cyctosine in the centre of the putative GC box were replaced by adenine in a manner analogous to the Sp1-specific mutation in phAR1.6Luc-UTRm1. Therefore, loss of Sp1 binding in this regulatory element is the dominant outcome, providing further evidence that the putative GC box can actively upregulate AR expression. In the likelihood that the substitution mutation of the ARS region was insufficient to prevent pur-α and hnRNP-K binding under physiological conditions, the entire regulatory element was deleted to create phAR1.6Luc-UTRΔm1. In this instance, promoter activity was upregulated by 1.27 and 1.67 fold in DU145 and LNCaP respectively (Fig 4B) with the difference between the two cell lines being significant (p<0.01).

Analogous mutations were also introduced into the third regulatory sequence of interest (+423 to +446). Firstly, the two GC boxes in Sp1-3 were mutated either individually (phAR1.6Luc-UTRm3 and phAR1.6Luc-UTRm4) or together (phAR1.6Luc-UTRm5). Luciferase activity revealed that disruption of the GC boxes resulted in significant decreases in promoter stimulation. The two single mutations decreased transcriptional activity by 42% and 29% in DU145 cells and by 29% and 17% in LNCaP cells, and the double mutation displayed reductions of 34% and 16% in the two cell lines respectively (Fig 4C). Again, the decreases in promoter activity were greater in DU145 than LNCaP. The effects of the mutations were not cumulative as might be expected from two overlapping binding sites. In a manner similar to that seen with the +322 to +345 region, base substitution mutation of the potential overlapping negative regulatory element in phAR1.6Luc-UTRm6 produced losses of transcriptional activity of 9% and 4% in DU145 and LNCaP respectively (Fig 4C). These values were less those seen with the +322 to +345 region described above, however, they were consistent with the sequence changes having only a minor impact on the Sp1 binding sites. Deletion of the entire potential negative regulatory element in phAR1.6Luc-UTRΔm2 led to 1.18 and 1.50 fold upregulation of transcriptional activity in DU145 and LNCaP respectively (Fig 4C). As with the phAR1.6Luc-UTRΔm1 deletion mutation, the increase in promoter activity was significantly greater in LNCaP than DU145.

### The binding characteristics of the 5’UTR overlapping regulatory elements

**Fig 5:**
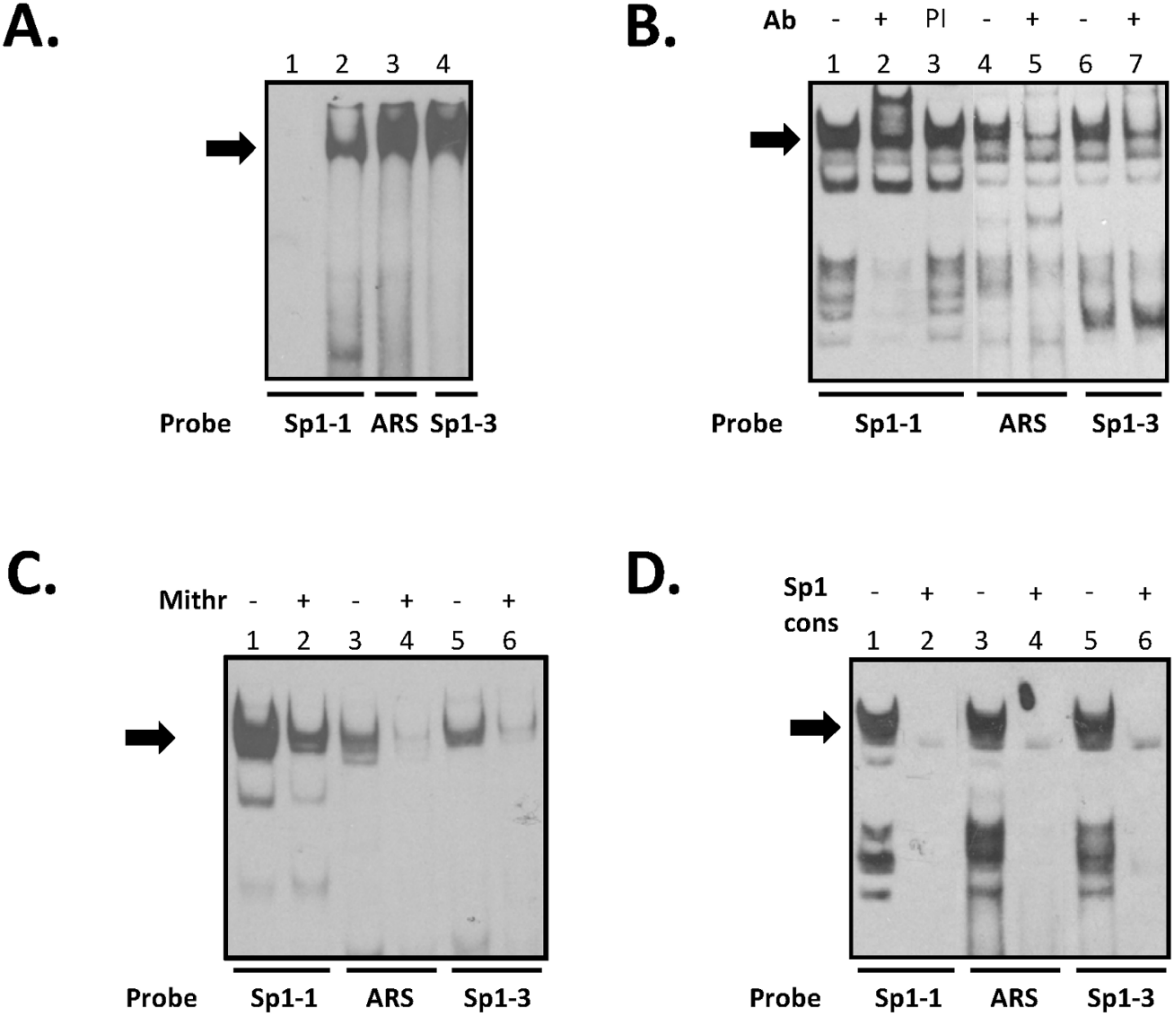
Binding of Sp1 to the AR gene promoter and 5’UTR sequences. Purified Sp1 protein (panel A) or nuclear extract from DU145 cells (panels (B) to (D)) were incubated with the labelled oligonucleotide probes indicated below each gel and the products resolved by electrophoretic mobility shift analysis. Additions are shown above the gels and were: Ab, antibody; PI, pre-immune serum; Mith, mithramycin; Sp1 cons, consensus Sp1 oligonucleotide. Competing unlabelled oligonucleotides (100 fold molar excess) or immune sera were added prior to addition of probe. EMSAs are representative of at least 3 independent experiments. (A) The indicated labelled probes were incubated with purified Sp1 protein except in lane 1 which had no addition. Black arrow indicates Sp1-DNA complex. (B) Anti-Sp1 antibody was added as indicated, and the complex absent after incubation with antibody is indicated by the arrow. (C) Mithramycin (120 nM) was added to indicated lanes and the complex depleted after incubation with the drug is indicated by the solid arrow. (D) Unlabelled competing oligonucleotide encoding Sp1 consensus binding site was added as shown.

The possibility that Sp1 binds to all three regions under study was examined by electrophoretic mobility shift assays (EMSAs). In initial experiments, purified recombinant human Sp1 protein was incubated with the labelled oligonucleotide probes containing either the primary GC box (Sp1-1), the potential novel Sp1 binding site in the ARS region or Sp1-3. Electrophoretic resolution of the resulting products with all three labelled probes showed a single high molecular weight DNA:protein complex near the top of the gel (Fig 5A, lanes 2–4) consistent with Sp1 having a molecular weight of 95 kDa.

Incubation of nuclear extract prepared from the prostate cancer cell line DU145 with either Sp1-1, ARS or Sp1-3 oligonucleotides produced several bands with virtually identical electrophoretic motilities (Fig 5B, lanes 1, 4 and 6 respectively). The binding of Sp1 was confirmed in all instances by the addition of anti-Sp1 antibody which produced a supershift with the Sp1-1 oligonucleotide and greatly diminished assembly of a high molecular weight DNA:protein complex with both ARS and Sp1-3 (Fig 5B, lanes 2, 5 and 7 respectively), while preimmune serum had no effect (Fig 5B, lane 3). Different outcomes of incubation with anti-transcription factor antibody can occur in EMSA analyses e.g. CREB-1 to CRE sites in the human insulin and somatostatin promoters [44], due to several factors including: the strength of binding to the DNA; the spatial/conformational organisation of the protein on regulatory elements, the orientation of DNA adjacent to the consensus and the presence of bound cofactors.

Addition of mithramycin A, which interferes with binding of Sp1 to its regulatory element [45], greatly reduced formation of the high molecular weight complexes with all three oligonucleotides (Fig 5C, compare lanes 1 and 2, 3 and 4, and 5 and 6). Similarly, preincubation with excess unlabelled oligonucleotide containing the consensus Sp1 binding sequence virtually eliminated all DNA:protein complex formation (Fig 5D, compare lanes 1 and 2, 3 and 4, and 5 and 6). Together, these results confirm binding of Sp1 to all three GC box-containing sequences, supporting the conclusions from reporter gene experiments (Fig 4). Further, the difference in results seen with the oligonucleotides for the Sp1-1 site in the core promoter in comparison to the ARS and Sp1-3 sites, which displayed apparent lower affinity for natural Sp1, different outcomes upon incubation with anti-Sp1 antibody and lower mutation-induced reduction in luciferase assays (Fig 4), suggested that binding of Sp1 to the core promoter GC box was stronger than to the 5’UTR.

**Fig 6:**
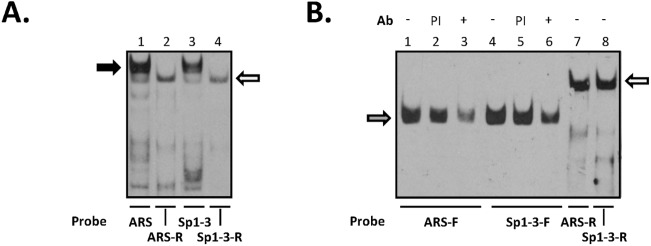
The AR 5’UTR possesses overlapping regulatory elements. Nuclear extract from DU145 cells were incubated with the labelled oligonucleotide probes indicated below each gel and the products resolved by electrophoretic mobility shift analysis. Additions are shown above the gels and were: Ab, antibody; PI, pre-immune serum. EMSAs are representative of at least 3 independent experiments. (A) Either double stranded probes or single stranded oligonucleotides of the reverse strand were incubated with nuclear extract and protein-DNA complexes resolved: black arrow indicates Sp1 and open arrow indicates pur-α binding respectively. (B) Forward and reverse single stranded labelled oligonucleotides of the regulatory elements were incubated with nuclear extract. Anti-hnRNP-K antibody was included where indicated and hnRNP-K-DNA complexes indicated by grey arrow and open arrow indicates pur-α binding.

The transcription factor pur-α binds preferentially to guanine-rich single stranded DNA or the equivalent sequences in double stranded DNA. Incubation of nuclear extract with either the double stranded or the G-rich single reverse strand of the ARS and Sp1-3 probes yielded quite dissimilar results. Both of the single stranded probes produced a DNA:protein complex (denoted by open arrow) that migrated slightly ahead of Sp1:DNA and other high molecular weight complexes typically seen with the double stranded probe (Fig 6A). Consistent with the ability of pur-α to also bind dsDNA, both the ARS and Sp1-3 double stranded oligonucleotides produced a faint DNA:protein complex that migrated in a very similar position to the product seen with the single stranded probes. Importantly, the DNA:protein complex formed with the G-rich strand of Sp1-3 behaved in an identical manner to that of the equivalent ARS oligonucleotide which is known to bind pur-α [36]. Interestingly, pur-α binds DNA as dimers and multimers [46] which would survive the non-denaturing conditions of EMSA resulting in the apparent high molecular weight migration characteristics of the DNA:protein complexes. The commercial anti-pur-α antibody failed to elicit an effect and the purified protein is not available. Nevertheless, the complementary cytosine-rich forward strands of the ARS and Sp1-3 oligonucleotides completely failed to produce the DNA:protein complex confirming the specificity of binding to the G-rich strand (Fig 6B; compare lanes 1 and 7, and 4 and 8).

The ubiquitous protein hnRNP-K, which binds to cytosine-rich single stranded RNA and DNA, also binds to the ARS in the hAR 5’UTR [36]. The binding of hnRNP-K to the C-rich forward strand of both the ARS and Sp1-3 sites was confirmed using anti-hnRNP-K antibody and the results are shown in Fig 6B. In both instances, a single DNA:protein complex was formed that migrated well ahead of the one seen previously with the G-rich reverse strand. Addition of preimmune serum had no effect on the formation of the DNA:protein complex, however, anti-hnRNP-K antibody greatly impeded the binding of the protein to the oligonucleotide probes.

### Sp1 binds to both 5’UTR regulatory elements

**Fig 7:**
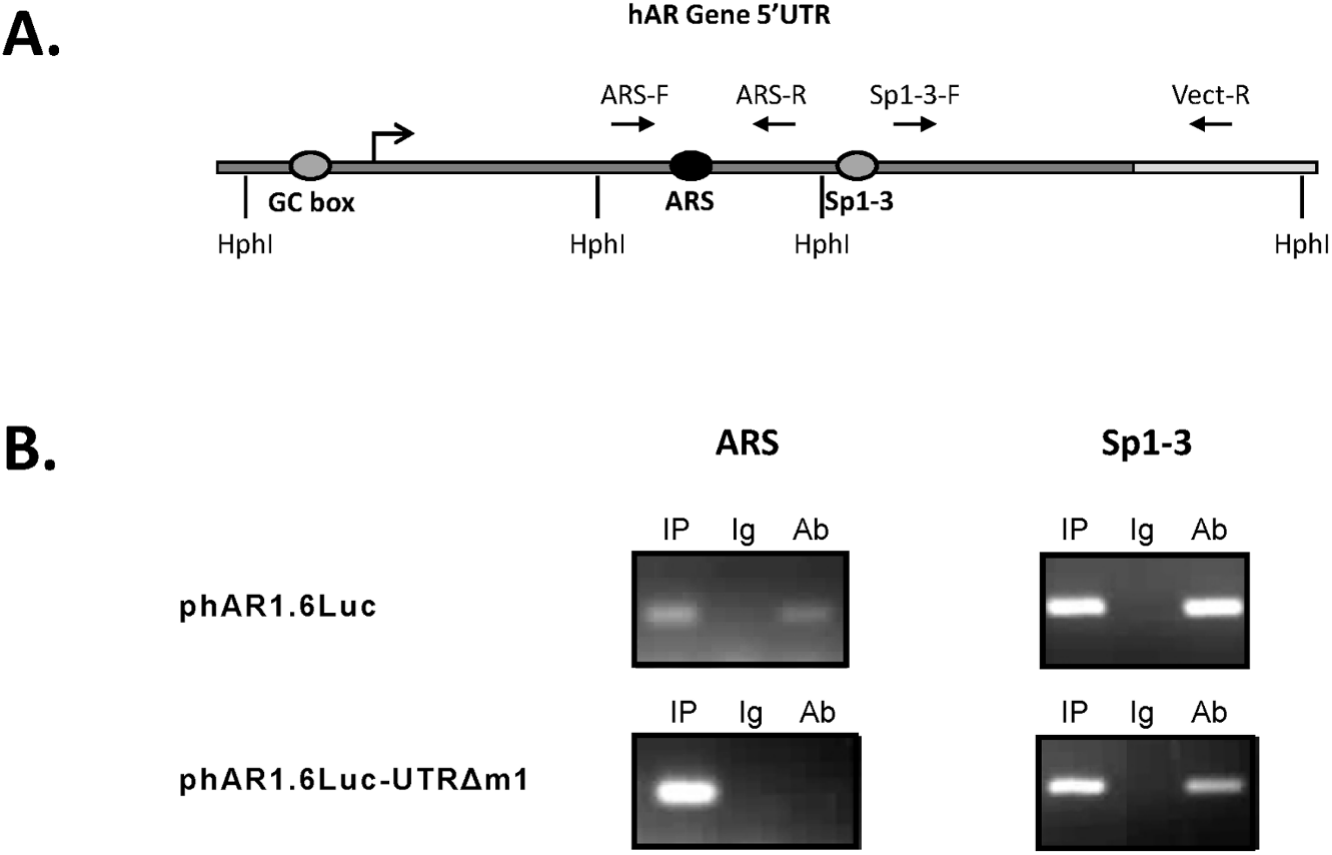
ChIP analysis confirms binding of Sp1 to 5’UTR ARS. (A) Line diagram (not to scale) of the hAR 5’UTR showing the recognition sites of the restriction endonuclease HphI used to digest plasmid, plus the forward (F) and reverse (R) primers (solid arrows) used for ChIP semi-quantitative PCR. Oligonucleotide Vect-R was specific for the plasmid vector sequences, and the bent arrow indicates the transcriptional start site. (B). Representative agarose gels of PCR amplified DNA immunoprecipitated with anti-Sp1 antibody in which the order of some lanes have been altered to aid clarity and facilitate comparisons; IP, input sample; Ig, pre-immune rabbit IgG; Ab, anti-Sp1 antibody.

In order to further validate the protein-DNA interactions, ChIP assays were undertaken to examine Sp1 binding to the two regions of interest in the hAR 5’UTR. The usual method of preparing chromatin for ChIP analysis involves sonication, however, as the ARS and Sp1-3 regulatory elements are only 100 bp apart, this methodology does not permit investigation of each of these regions in isolation. Therefore, restriction enzyme digestion of chromatin was employed instead. The only commercially available enzyme that cut the DNA between the different Sp1 binding sites without complications from possible CpG methylation in the very high GC rich hAR promoter and 5’UTR was HphI. As commercially available preparations of HphI were not of high enough concentrations to yield sufficient levels of solubilised nuclear chromatin, LNCaP cells were transiently transfected with phAR1.6Luc (WT) or phAR1.6Luc-UTRΔm1. The plasmids were digested with HphI and the solubilised DNA was precipitated using anti-Sp1 antibody. Fig 7 depicts the hAR 5’UTR along with the cleavage sites for HphI and the relative positions of the oligonucleotides used for semi-quantitative PCR amplification.

The Sp1-3 region served as a positive control and anti-Sp1 antibody, but not preimmune Ig immunoprecipitated both the ARS and Sp1-3 regions of the 5’UTR, confirming that Sp1 binds to the ARS (Fig 5B). Conversely, phAR1.6Luc-Δm1, in which the ARS regulatory element had been deleted, was not precipitated by anti-Sp1 antibody. This result also indicated that the action of the antibody was specific.

### The relative amounts of Sp1 and pur-α influence the activity of the overlapping regulatory elements

**Fig 8:**
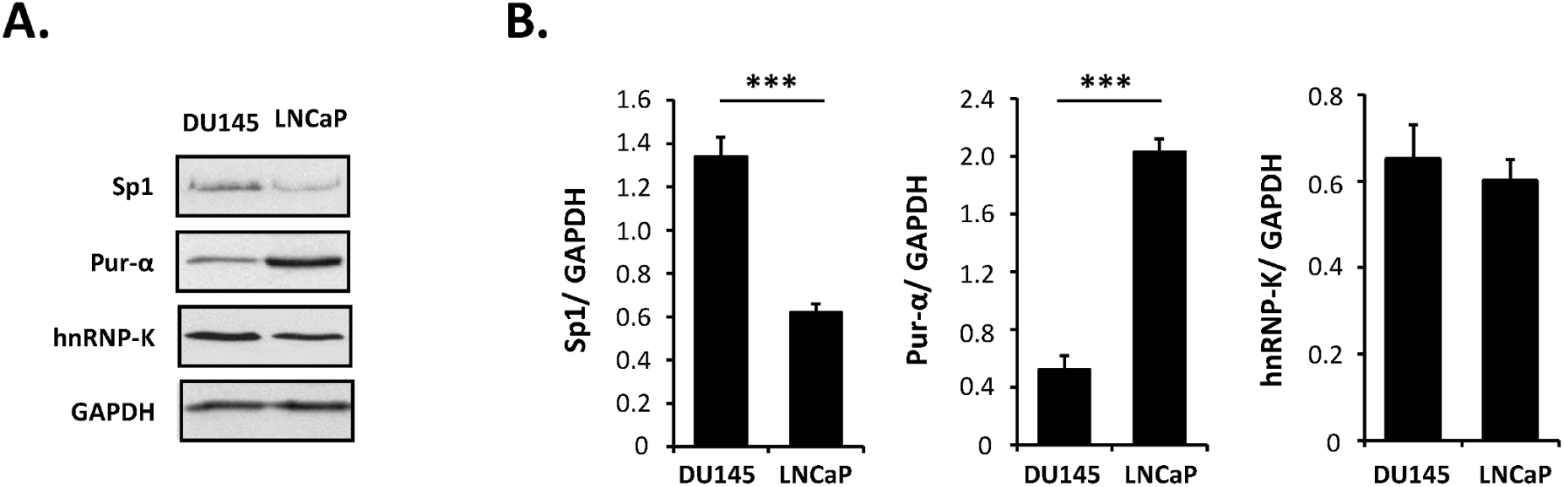
Variation of transcription factor levels in PCa cells. Western blot analysis of Sp1, pur-α and hnRNP-K protein levels normalised against GAPDH in the indicated PCa cell lines. The data represent the means ± SEM of at least three independent experiments and statistical significance is: *** p<0.001.

The possibility that the different outcomes of mutation of the Sp1 and pur-α binding sites in DU145 and LNCaP could be directed by aberrant cell signalling was examined by comparing the levels of the associated transcription factors using western blot analysis. Relative to the house-keeping enzyme GAPDH, DU145 cells had 2.2 fold higher levels of Sp1 in comparison to LNCaP while the converse occurred in the case of pur-α with LNCaP cells having 3.9 fold higher levels in comparison to DU145 (Fig 8). These results are in agreement with other reports that pur-α is decreased at both the protein and mRNA levels in PC3 and DU145 compared to LNCaP cells [47], while the levels of Sp1 were elevated in DU145 compared with LNCaP. No significant difference was noted in the levels of hnRNP-K between the two PCa cell lines.

Western blot analyses reveals striking differences in the relative amounts of Sp1 and pur-α in the two PCa cell lines. The ratios of transcription factors Sp1 to pur-α are consistent with the promoter activity observed in the luciferase assays. Mutation of the dominant GC box in the promoter (Sp1-1), which binds only Sp1 and not pur-α, reduced transcriptional activity to a significantly greater degree in LNCaP than DU145 which is compatible with the former cell type having much lower levels of Sp1 and thus having lower capacity to compensate for the impaired binding to the regulatory element in the promoter, or through compensatory binding to GC boxes in the 5’UTR. A different scenario was evident at the 5’UTR ARS and Sp1-3 sites where stimulatory Sp1 and inhibitory pur-α were in competition. In both instances, mutation of the Sp1-binding GC boxes resulted in a loss of activity implying that, in the cells used in the studies, the balance of up—and downregulation tended towards Sp1 stimulation. The effects of mutation were more pronounced in DU145, which had an almost 9 fold greater ratio of Sp1 to pur-α, whereas in LNCaP with higher levels of pur-α the upregulatory role of the two 5’UTR WT regions would have been lower. Similarly, complete deletion of the regions to ensure no binding of either Sp1 or pur-α led to significantly greater release of inhibition in pur-α-rich LNCaP than in DU145.

## Discussion

**Fig 9:**
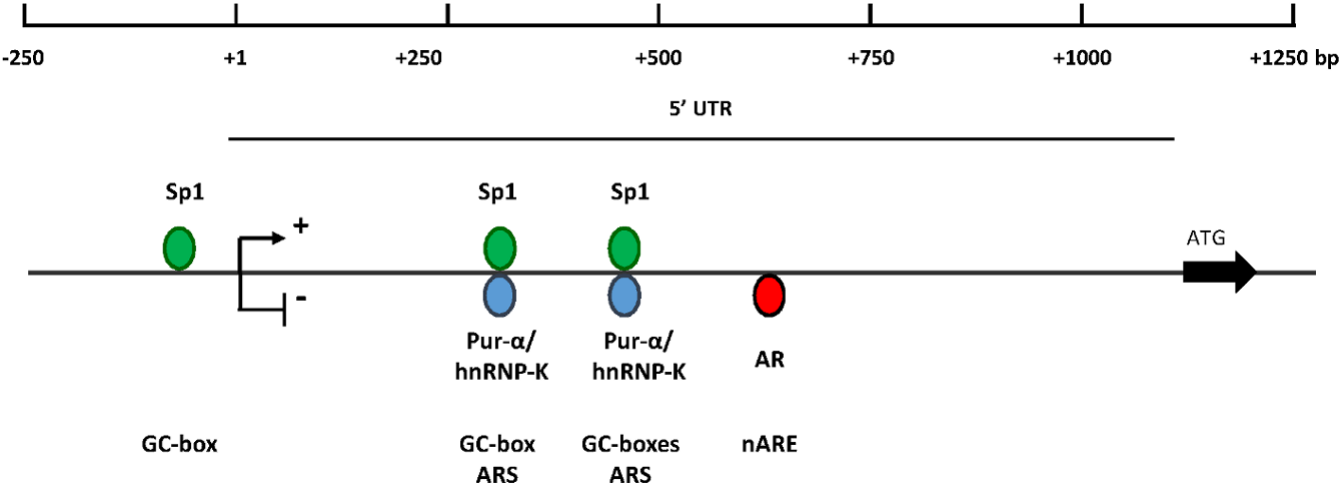
Model for regulation of the human AR gene. The organisation of positive (GC-box) and negative (ARS, nARE) elements in the promoter and 5’UTR of the AR gene are shown together with the relevant transcription factors.

High levels of androgen receptor expression are often a crucial factor in driving progression of PCa leading to resistance to ADT and drug regimes. In order to control AR expression, a more detailed understanding of the intricacies of human AR gene regulation is of fundamental importance. Consequently, this study focussed on the role of the primary driver of AR expression; namely, the transcription factor Sp1 and its binding sites. The GC box in the promoter immediately upstream of the start of transcription functions was determined to be the principal Sp1 binding site, although it is not absolutely necessary as additional GC boxes in the 5’UTR-coding region also participate in stimulation of AR expression; albeit at a lower level. This implies that the hAR promoter and 5’UTR have a flexible configuration permitting dynamic remodelling to create the initiation complex. In addition, two novel regulatory elements were identified in the 5’UTR; an upregulatory GC box and an inhibitory ARS (Fig 9). Importantly, in both cases the upregulatory GC boxes overlapped inhibitory ARS regions and the transcriptional outcome in PCa cells was determined by the relative amounts of the associated antagonistic transcription factors Sp1 and pur-α (Fig 8).

Sp1 belongs to the highly conserved Specificity Protein/Krüppel-like Factor (SP/KLF) transcription factor family and is expressed in all mammalian cells (reviewed in [32]). Although it was originally considered to function primarily as a constitutive activator of TATA-less promoters e.g. those of housekeeping genes, this view has undergone a radical shift with Sp1 also participating in more nuanced gene regulation [32]. Importantly, Sp1 is elevated in many cancers [32,48] and studies using LNCaP cells show that this leads to increased AR transcription [49]. On the other hand, the inhibitory ARS sites that overlap the GC boxes in the two studied regions can bind the ubiquitously expressed protein pur-α with increased pur-α expression through the release of epigenic repression in androgen insensitive LNCaP cells leading to reduced AR expression and concomitant cell proliferation [36], as well as increased sensitivity to bicalutamide [50].

The physical properties of Sp1, pur-α and hnRNP-K mean that their roles in regulating hAR transcription can be multi-faceted. Sp1 has the ability to form multimers producing DNA looping that is discernible by electron microscopy [51] thus bringing regulatory elements and enhancers together. Further levels of complexity are generated by the capacity of Sp1 to associate with pur-α [52] which can in turn form multimeric complexes with a range of other proteins [53]. Genome-wide studies reveal co-localisation of Sp1 motifs and AR binding sites suggesting interplay between these proteins and their signalling pathways [54], and it is interesting to note that AR binds to the hAR 5’UTR [34] distal to the regions described here. Sp1 can also interact directly with AR, and in the case of the c-Met promoter in PCa cells this reduces its upregulatory action [55]. Taken together, the overlapping regulatory elements in the hAR 5’UTR described here have the ability to not only influence transcription through the relative binding of competing Sp1 and pur-α transcription factors, but to participate in a dynamic interplay between multiple proteins to produce numerous outcomes that are heavily influenced by the confluence of diverse cell signalling pathways.

The results of this study highlight the paramount role of Sp1 in driving hAR expression in PCa cells and, therefore, the appropriateness of targeting Sp1 as a means of suppressing tumour progression. A diverse range of both synthetic and natural agents that downregulate Sp1 induced AR expression have been shown to be efficacious in a variety PCa cell line and animal model systems. The anti-tumour antibiotic mithramycin A [29], recent analogues with improved pharmacological and toxicological properties [56], phenethyl isothiocyanate (PEITC) present in cruciferous vegetables [57,58] and acetyl-11-keto-beta-boswellic acid (AKBA) from *Boswellia serrata* [59] act by disrupting Sp1 binding to GC-boxes, while betulinic acid from white birch bark [60] and green tea phenols [61] reduce the levels of Sp1 or transactivation activity. However, the finding that DHT-bound AR can also inhibit Sp1 expression [62] may be a confounding factor when using anti-androgen therapies, as this would be predicted to lead to elevated Sp1 levels.

There is considerable interest in the mechanisms that control the levels of the AR protein in different cells and pathophysiological conditions. Our identification of overlapping regulatory elements that bind antagonistic transcription factors may facilitate rapid changes in expression of the receptor in response to the relative amounts of Sp1 and pur-α. Reduction of Sp1 or inhibition of its action through the GC boxes detailed here will not only lead to a decrease in AR mRNA directly, but will also promote the binding of inhibitory pur-α to the super-imposed ARS sites. This synergistic effect will be especially advantageous in CRPC as pur-α levels are frequently reduced [36].

## Supporting Information

S1 Fig Alignment of the human AR 5’UTR and the mouse suppressor element. (A). Mouse AR 5’UTR suppressor element protected from DNase I digestion [40] with the confirmed human ARS (both blue underlined). (B) Mouse AR 5’UTR suppressor element as defined by [41] (blue underlined) with the confirmed human GC boxes (green underlined). (C). Mouse AR 5’UTR suppressor element as defined by [41] and the confirmed human ARS (both blue underlined). Homologous sequences are demarked by vertical lines.
(TIF)

S2 Fig Multiple genome alignments against the mouse AR 5’UTR ARS. The region of the mouse AR gene 5’UTR encoding the ARS (blue box) was compared to those of the indicated placental species. Differences from the mouse sequence are indicated by bold, underlined font.
(TIF)

S1 File Table A. Oligonucleotides used in creating phAR1.6Luc substitution mutations. Table B. Oligonucleotides used in creating phAR1.6Luc deletion mutations.
(DOCX)

## References

[1] WHO. Cancer Fact Sheet, GLOBOCAN 2008. 2008.

[2] Center MM, Jemal A, Lortet-Tieulent J, Ward E, Ferlay J, Brawley O, et al International variation in prostate cancer incidence and mortality rates. Eur Urol. 2012;61: 1079–1092. 10.1016/j.eururo.2012.02.054 22424666

[3] Heinlein CA, Chang C. Androgen receptor in prostate cancer. Endocr Rev. 2004;25: 276–308. 15082523 10.1210/er.2002-0032

[4] Dehm SM, Tindall DJ. Androgen receptor structural and functional elements: role and regulation in prostate cancer. Mol Endocrinol. 2007;21: 2855–2863. 17636035 10.1210/me.2007-0223

[5] Lonergan PE, Tindall DJ. Androgen receptor signaling in prostate cancer development and progression. J Carcinog. 2011;10: 20–3163. 83937. Epub 2011 Aug 23. 10.4103/1477-3163.83937 21886458 PMC3162670

[6] Shen MM, Abate-Shen C. Molecular genetics of prostate cancer: new prospects for old challenges. Genes Dev. 2010;24: 1967–2000. 10.1101/gad.1965810 20844012 PMC2939361

[7] Azzouni F, Mohler J. Biology of castration-recurrent prostate cancer. Urol Clin North Am. 2012;39: 435–452. 10.1016/j.ucl.2012.07.002 23084522

[8] Scher HI, Sawyers CL. Biology of progressive, castration-resistant prostate cancer: directed therapies targeting the androgen-receptor signaling axis. J Clin Oncol. 2005;23: 8253–8261. 16278481 10.1200/JCO.2005.03.4777

[9] Green SM, Mostaghel EA, Nelson PS. Androgen action and metabolism in prostate cancer. Mol Cell Endocrinol. 2012;360: 3–13. 10.1016/j.mce.2011.09.046 22453214 PMC4124858

[10] Tararova ND, Narizhneva N, Krivokrisenko V, Gudkov AV, Gurova KV. Prostate cancer cells tolerate a narrow range of androgen receptor expression and activity. Prostate. 2007;67: 1801–1815. 17935158 10.1002/pros.20662 PMC2914504

[11] Wu D, Zhang C, Shen Y, Nephew KP, Wang Q. Androgen receptor-driven chromatin looping in prostate cancer. Trends Endocrinol Metab. 2011;22: 474–480. 10.1016/j.tem.2011.07.006 21889355 PMC3229688

[12] Heemers HV, Tindall DJ. Androgen receptor (AR) coregulators: a diversity of functions converging on and regulating the AR transcriptional complex. Endocr Rev. 2007;28: 778–808. 17940184 10.1210/er.2007-0019

[13] He B, Lanz RB, Fiskus W, Geng C, Yi P, Hartig SM, et al GATA2 facilitates steroid receptor coactivator recruitment to the androgen receptor complex. Proc Natl Acad Sci U S A. 2014;111: 18261–18266. 10.1073/pnas.1421415111 25489091 PMC4280633

[14] Wu D, Sunkel B, Chen Z, Liu X, Ye Z, Li Q, et al Three-tiered role of the pioneer factor GATA2 in promoting androgen-dependent gene expression in prostate cancer. Nucleic Acids Res. 2014;42: 3607–3622. 10.1093/nar/gkt1382 24423874 PMC3973339

[15] Shah S, Prasad S, Knudsen KE. Targeting pioneering factor and hormone receptor cooperative pathways to suppress tumor progression. Cancer Res. 2012;72: 1248–1259. 10.1158/0008-5472.CAN-11-0943 22258452 PMC3294022

[16] Chen Y, Sawyers CL, Scher HI. Targeting the androgen receptor pathway in prostate cancer. Curr Opin Pharmacol. 2008;8: 440–448. 10.1016/j.coph.2008.07.005 18674639 PMC2574839

[17] Latil A, Bieche I, Vidaud D, Lidereau R, Berthon P, Cussenot O, et al Evaluation of androgen, estrogen (ER alpha and ER beta), and progesterone receptor expression in human prostate cancer by real-time quantitative reverse transcription-polymerase chain reaction assays. Cancer Res. 2001;61: 1919–1926. 11280747

[18] Linja MJ, Savinainen KJ, Saramaki OR, Tammela TL, Vessella RL, Visakorpi T. Amplification and overexpression of androgen receptor gene in hormone-refractory prostate cancer. Cancer Res. 2001;61: 3550–3555. 11325816

[19] Li TH, Zhao H, Peng Y, Beliakoff J, Brooks JD, Sun Z. A promoting role of androgen receptor in androgen-sensitive and -insensitive prostate cancer cells. Nucleic Acids Res. 2007;35: 2767–2776. 17426117 10.1093/nar/gkm198 PMC1885678

[20] Carreira S, Romanel A, Goodall J, Grist E, Ferraldeschi R, Miranda S, et al Tumor clone dynamics in lethal prostate cancer. Sci Transl Med. 2014;6: 254ra125 10.1126/scitranslmed.3009448 25232177 PMC4422178

[21] Urbanucci A, Sahu B, Seppala J, Larjo A, Latonen LM, Waltering KK, et al Overexpression of androgen receptor enhances the binding of the receptor to the chromatin in prostate cancer. Oncogene. 2012;31: 2153–2163. 10.1038/onc.2011.401 21909140

[22] Waltering KK, Helenius MA, Sahu B, Manni V, Linja MJ, Janne OA, et al Increased expression of androgen receptor sensitizes prostate cancer cells to low levels of androgens. Cancer Res. 2009;69: 8141–8149. 10.1158/0008-5472.CAN-09-0919 19808968

[23] Decker KF, Zheng D, He Y, Bowman T, Edwards JR, Jia L. Persistent androgen receptor-mediated transcription in castration-resistant prostate cancer under androgen-deprived conditions. Nucleic Acids Res. 2012;40: 10765–10779. 10.1093/nar/gks888 23019221 PMC3510497

[24] Wang Q, Li W, Zhang Y, Yuan X, Xu K, Yu J, et al Androgen receptor regulates a distinct transcription program in androgen-independent prostate cancer. Cell. 2009;138: 245–256. 10.1016/j.cell.2009.04.056 19632176 PMC2726827

[25] Sharma NL, Massie CE, Ramos-Montoya A, Zecchini V, Scott HE, Lamb AD, et al The androgen receptor induces a distinct transcriptional program in castration-resistant prostate cancer in man. Cancer Cell. 2013;23: 35–47. 10.1016/j.ccr.2012.11.010 23260764

[26] Palmberg C, Koivisto P, Hyytinen E, Isola J, Visakorpi T, Kallioniemi OP, et al Androgen receptor gene amplification in a recurrent prostate cancer after monotherapy with the nonsteroidal potent antiandrogen Casodex (bicalutamide) with a subsequent favorable response to maximal androgen blockade. Eur Urol. 1997;31: 216–219. 9076469 10.1159/000474453

[27] Takane KK, McPhaul MJ. Functional analysis of the human androgen receptor promoter. Mol Cell Endocrinol. 1996;119: 83–93. 8793857 10.1016/0303-7207(96)03800-2

[28] Faber PW, van Rooij HC, Schipper HJ, Brinkmann AO, Trapman J. Two different, overlapping pathways of transcription initiation are active on the TATA-less human androgen receptor promoter. The role of Sp1. J Biol Chem. 1993;268: 9296–9301. 8486625

[29] Wang LG, Ferrari AC. Mithramycin Targets Sp1 and The Androgen Receptor Transcription Level—Potential Therapeutic Role in Advanced Prostate Cancer. 2006;2: 19–31. PMC3642134 23662037

[30] Chen S, Supakar PC, Vellanoweth RL, Song CS, Chatterjee B, Roy AK. Functional role of a conformationally flexible homopurine/homopyrimidine domain of the androgen receptor gene promoter interacting with Sp1 and a pyrimidine single strand DNA-binding protein. Mol Endocrinol. 1997;11: 3–15. 8994183 10.1210/mend.11.1.9868

[31] Courey AJ, Tjian R. Analysis of Sp1 in vivo reveals multiple transcriptional domains, including a novel glutamine-rich activation motif. Cell. 1988;55: 887–898. 3142690 10.1016/0092-8674(88)90144-4

[32] Li L, Davie JR. The role of Sp1 and Sp3 in normal and cancer cell biology. Ann Anat. 2010;192: 275–283. 10.1016/j.aanat.2010.07.010 20810260

[33] Ko S, Shi L, Kim S, Song CS, Chatterjee B. Interplay of nuclear factor-kappaB and B-myb in the negative regulation of androgen receptor expression by tumor necrosis factor alpha. Mol Endocrinol. 2008;22: 273–286. 17975021 10.1210/me.2007-0332 PMC2234585

[34] Hay CW, Watt K, Hunter I, Lavery DN, MacKenzie A, McEwan IJ. Negative Regulation of the Androgen Receptor Gene Through a Primate-Specific Androgen Response Element Present in the 5' UTR. Horm Cancer. 2014. 10.1007/s12672-014-0185-y PMC4164857 24895212

[35] Wang LG, Ossowski L, Ferrari AC. Androgen receptor level controlled by a suppressor complex lost in an androgen-independent prostate cancer cell line. Oncogene. 2004;23: 5175–5184. 15156193 10.1038/sj.onc.1207654

[36] Wang LG, Johnson EM, Kinoshita Y, Babb JS, Buckley MT, Liebes LF, et al Androgen receptor overexpression in prostate cancer linked to Pur alpha loss from a novel repressor complex. Cancer Res. 2008;68: 2678–2688. 10.1158/0008-5472.CAN-07-6017 18413735

[37] Chen Z, Lan X, Thomas-Ahner JM, Wu D, Liu X, Ye Z, et al Agonist and antagonist switch DNA motifs recognized by human androgen receptor in prostate cancer. EMBO J. 2015;34: 502–516. 10.15252/embj.201490306 25535248 PMC4331004

[38] Hay CW, McEwan IJ. The impact of point mutations in the human androgen receptor: classification of mutations on the basis of transcriptional activity. PLoS One. 2012;7: e32514 10.1371/journal.pone.0032514 22403669 PMC3293822

[39] Dignam JD, Lebovitz RM, Roeder RG. Accurate transcription initiation by RNA polymerase II in a soluble extract from isolated mammalian nuclei. Nucleic Acids Res. 1983;11: 1475–1489. 6828386 10.1093/nar/11.5.1475 PMC325809

[40] Grossmann ME, Lindzey J, Kumar MV, Tindall DJ. The mouse androgen receptor is suppressed by the 5'-untranslated region of the gene. Mol Endocrinol. 1994;8: 448–455. 8052266 10.1210/mend.8.4.8052266

[41] Grossmann ME, Tindall DJ. The androgen receptor is transcriptionally suppressed by proteins that bind single-stranded DNA. J Biol Chem. 1995;270: 10968–10975. 7738038 10.1074/jbc.270.18.10968

[42] Steiper ME, Young NM. Primate molecular divergence dates. Mol Phylogenet Evol. 2006;41: 384–394. 16815047 10.1016/j.ympev.2006.05.021

[43] Mizokami A, Yeh SY, Chang C. Identification of 3',5'-cyclic adenosine monophosphate response element and other cis-acting elements in the human androgen receptor gene promoter. Mol Endocrinol. 1994;8: 77–88. 8152432 10.1210/mend.8.1.8152432

[44] Hay CW, Ferguson LA, Docherty K. ATF–2 stimulates the human insulin promoter through the conserved CRE2 sequence. Biochim Biophys Acta. 2007;1769: 79–91. 17337306 10.1016/j.bbaexp.2007.01.005

[45] Snyder RC, Ray R, Blume S, Miller DM. Mithramycin blocks transcriptional initiation of the c-myc P1 and P2 promoters. Biochemistry. 1991;30: 4290–4297. 1827033 10.1021/bi00231a027

[46] Johnson EM, Daniel DC, Gordon J. The pur protein family: genetic and structural features in development and disease. J Cell Physiol. 2013;228: 930–937. 10.1002/jcp.24237 23018800 PMC3747735

[47] Inoue T, Leman ES, Yeater DB, Getzenberg RH. The potential role of purine-rich element binding protein (PUR) alpha as a novel treatment target for hormone-refractory prostate cancer. Prostate. 2008;68: 1048–1056. 10.1002/pros.20764 18386260

[48] Sankpal UT, Goodison S, Abdelrahim M, Basha R. Targeting Sp1 transcription factors in prostate cancer therapy. Med Chem. 2011;7: 518–525. 22022994 10.2174/157340611796799203

[49] Yuan H, Gong A, Young CY. Involvement of transcription factor Sp1 in quercetin-mediated inhibitory effect on the androgen receptor in human prostate cancer cells. Carcinogenesis. 2005;26: 793–801. 15661808 10.1093/carcin/bgi021

[50] Liu X, Gomez-Pinillos A, Liu X, Johnson EM, Ferrari AC. Induction of bicalutamide sensitivity in prostate cancer cells by an epigenetic Puralpha-mediated decrease in androgen receptor levels. Prostate. 2010;70: 179–189. 10.1002/pros.21051 19790234

[51] Su W, Jackson S, Tjian R, Echols H. DNA looping between sites for transcriptional activation: self-association of DNA-bound Sp1. Genes Dev. 1991;5: 820–826. 1851121 10.1101/gad.5.5.820

[52] Tretiakova A, Steplewski A, Johnson EM, Khalili K, Amini S. Regulation of myelin basic protein gene transcription by Sp1 and Puralpha: evidence for association of Sp1 and Puralpha in brain. J Cell Physiol. 1999;181: 160–168. 10457364 10.1002/(SICI)1097-4652(199910)181:1<160::AID-JCP17>3.0.CO;2-H

[53] White MK, Johnson EM, Khalili K. Multiple roles for Puralpha in cellular and viral regulation. Cell Cycle. 2009;8: 1–7. 19182532 10.4161/cc.8.3.7585 PMC2683411

[54] Tewari AK, Yardimci GG, Shibata Y, Sheffield NC, Song L, Taylor BS, et al Chromatin accessibility reveals insights into androgen receptor activation and transcriptional specificity. Genome Biol. 2012;13: R88 10.1186/gb-2012-13-10-r88 23034120 PMC3491416

[55] Verras M, Lee J, Xue H, Li TH, Wang Y, Sun Z. The androgen receptor negatively regulates the expression of c-Met: implications for a novel mechanism of prostate cancer progression. Cancer Res. 2007;67: 967–975. 17283128 10.1158/0008-5472.CAN-06-3552

[56] Malek A, Nunez LE, Magistri M, Brambilla L, Jovic S, Carbone GM, et al Modulation of the activity of Sp transcription factors by mithramycin analogues as a new strategy for treatment of metastatic prostate cancer. PLoS One. 2012;7: e35130 10.1371/journal.pone.0035130 22545098 PMC3334962

[57] Wang LG, Liu XM, Chiao JW. Repression of androgen receptor in prostate cancer cells by phenethyl isothiocyanate. Carcinogenesis. 2006;27: 2124–2132. 16704988 10.1093/carcin/bgl075

[58] Beklemisheva AA, Feng J, Yeh YA, Wang LG, Chiao JW. Modulating testosterone stimulated prostate growth by phenethyl isothiocyanate via Sp1 and androgen receptor down-regulation. Prostate. 2007;67: 863–870. 17431886 10.1002/pros.20472

[59] Yuan HQ, Kong F, Wang XL, Young CY, Hu XY, Lou HX. Inhibitory effect of acetyl-11-keto-beta-boswellic acid on androgen receptor by interference of Sp1 binding activity in prostate cancer cells. Biochem Pharmacol. 2008;75: 2112–2121. 10.1016/j.bcp.2008.03.005 18430409

[60] Chintharlapalli S, Papineni S, Ramaiah SK, Safe S. Betulinic acid inhibits prostate cancer growth through inhibition of specificity protein transcription factors. Cancer Res. 2007;67: 2816–2823. 17363604 10.1158/0008-5472.CAN-06-3735

[61] Ren F, Zhang S, Mitchell SH, Butler R, Young CY. Tea polyphenols down-regulate the expression of the androgen receptor in LNCaP prostate cancer cells. Oncogene. 2000;19: 1924–1932. 10773882 10.1038/sj.onc.1203511

[62] Song K, Wang H, Krebs TL, Kim SJ, Danielpour D. Androgenic control of transforming growth factor-beta signaling in prostate epithelial cells through transcriptional suppression of transforming growth factor-beta receptor II. Cancer Res. 2008;68: 8173–8182. 10.1158/0008-5472.CAN-08-2290 18829577 PMC2596934

