# Supplementary figures and images for: An Sp1 Modulated Regulatory Region Unique to Higher Primates Regulates Human Androgen Receptor Promoter Activity in Prostate Cancer Cells
Source: PLoS One. 2015 Oct 8;10(10):e0139990. doi: 10.1371/journal.pone.0139990 (PMC4598089; doi:10.1371/journal.pone.0139990)

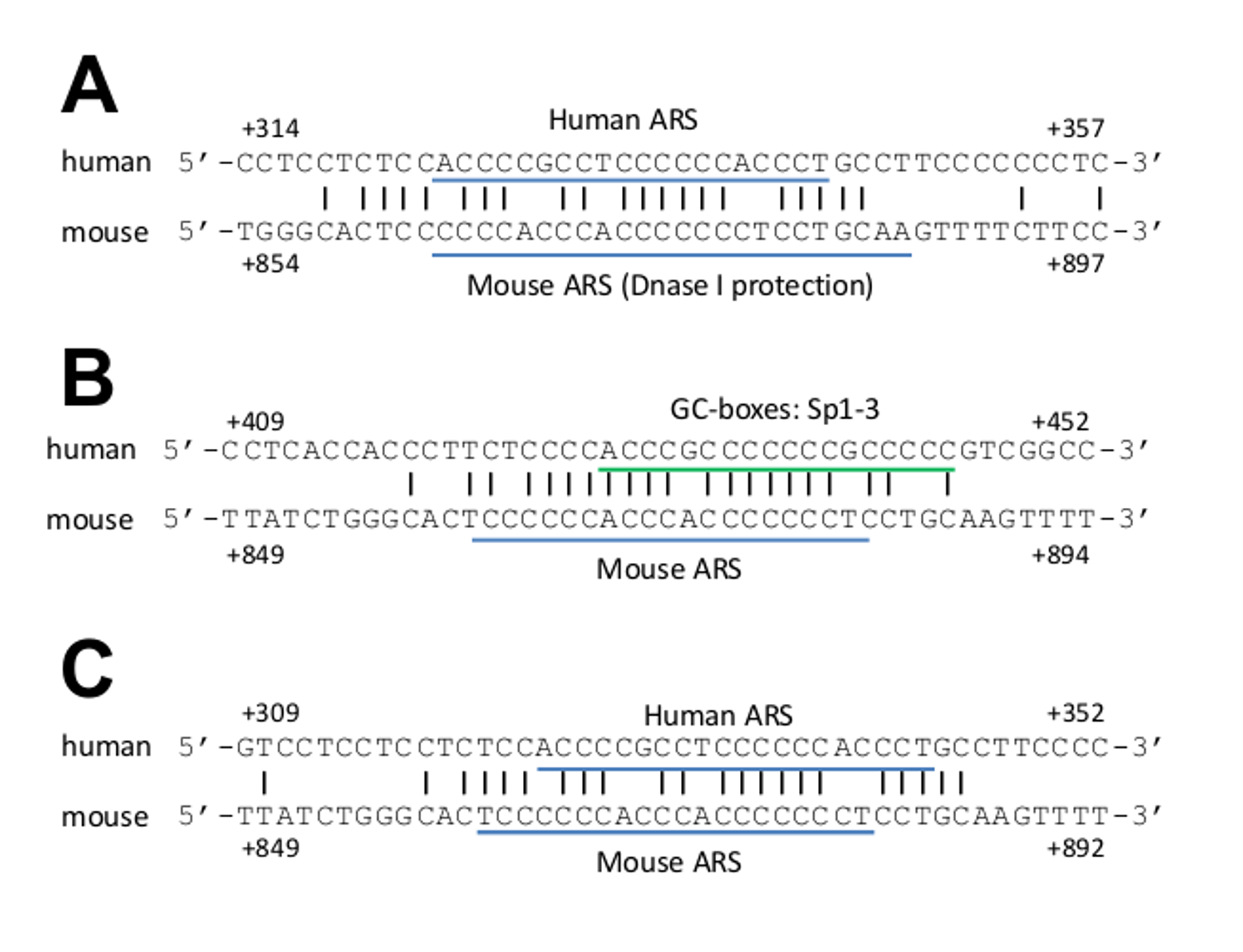

Supplement: S1 Fig — (A). Mouse AR 5’UTR suppressor element protected from DNase I digestion [40] with the confirmed human ARS (both blue underlined). (B) Mouse AR 5’UTR suppressor element as defined by [41] (blue underlined) with the confirmed human GC boxes (green underlined). (C). Mouse AR 5’UTR suppressor element as defined by [41] and the confirmed human ARS (both blue underlined). Homologous sequences are demarked by vertical lines. (TIF) [file pone.0139990.s001.tif]

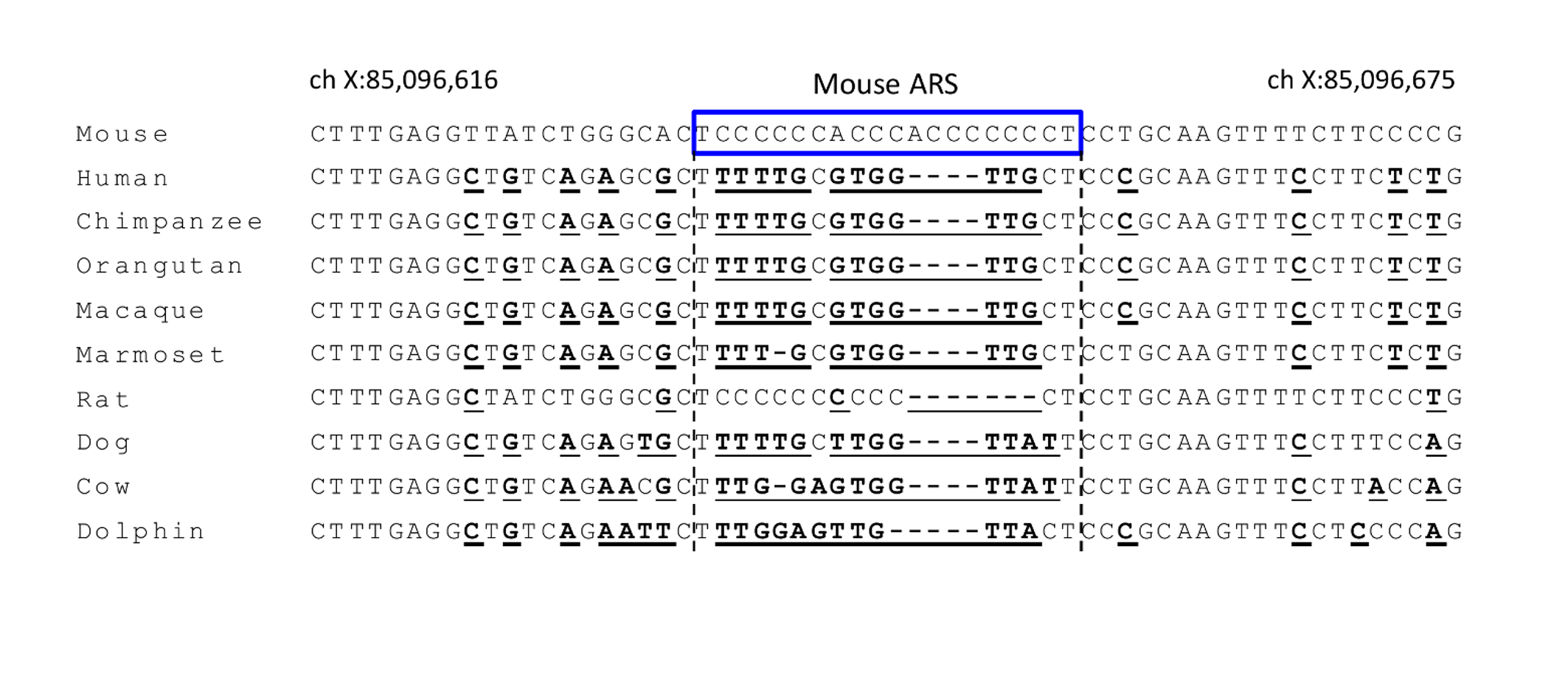

Supplement: S2 Fig — The region of the mouse AR gene 5’UTR encoding the ARS (blue box) was compared to those of the indicated placental species. Differences from the mouse sequence are indicated by bold, underlined font. (TIF) [file pone.0139990.s002.tif]
