# Supplementary material for: An Sp1 Modulated Regulatory Region Unique to Higher Primates Regulates Human Androgen Receptor Promoter Activity in Prostate Cancer Cells
Source: PLoS One. 2015 Oct 8;10(10):e0139990. doi: 10.1371/journal.pone.0139990 (PMC4598089; doi:10.1371/journal.pone.0139990)
Supplement: S1 File — Table A. Oligonucleotides used in creating phAR1.6Luc substitution mutations. Table B. Oligonucleotides used in creating phAR1.6Luc deletion mutations. (DOCX) [file pone.0139990.s003.docx]

**Supplementary Information**

Table A **Oligonucleotides used in creating phAR1.6Luc substitution mutations.** Mutated bases shown in bold font.

| Mutation | Oligonucleotide |
| --- | --- |
| phAR1.6Luc‑ARSm1 | 5’‑TCCTCCTCCTCTCCACC**AAAA**CTCCCCCCACCCTGCC‑3’ |
| phAR1.6Luc‑UTRm2 | 5’‑CCTCCTCTCCACCC**AA**CCTCC**TT**CCACCCTGCCTTCC‑3’ |
| phAR1.6Luc‑UTRm3 | 5’‑CCACCCTTCTCCCCACC**AAAA**CCCCCGCCCCCGTCG‑3’ |
| phAR1.6Luc‑UTRm4 | 5’‑CCCACCCGCCCCCC**AAAA**CCCGTCGGCCCAGCG‑3 |
| phAR1.6Luc‑UTRm5 (by mutation of phAR1.6Luc‑UTRm4) | 5’‑CCACCCTTCTCCCCACC**AAAA**CCCCAAAACCCGTCGGC‑3’ |
| phAR1.6Luc‑UTRm6 | 5’‑CACCCTTCTCCC**A**ACCCGC**TT**CCCCGCCCCCG‑3’ |

Table A2 **Oligonucleotides used in creating phAR1.6Luc deletion mutations.**

| Mutation | Oligonucleotide |
| --- | --- |
| vector‑F | 5’‑CCGGTACCTGAGCTCTGGACAAAATTGAG‑3’ |
| vector‑R | 5’‑GAGCTCAGGTACCGGCCAGTTAGG‑3’ |
| Δm1‑F | 5’‑GTCCTCCTCCTCTCCGCCTTCCCCCCCTCCCCCGT‑3 |
| Δm1‑R | 5’‑GGAGAGGAGGAGGACAAAGGCAGC‑3’ |
| Δm2‑F | 5’‑CCTCACCACCCTTCTCCCGTCGGCCCAGCGCTGC‑3’ |
| Δm2‑R | 5’‑AGAAGGGTGGTGAGGGGGGTTGG‑3’ |
